# Congenital deformity of the paw in a captive tiger: case report

**DOI:** 10.1186/1746-6148-8-98

**Published:** 2012-06-29

**Authors:** Sheila C Rahal, Reinaldo S Volpi, Carlos R Teixeira, Vania MV Machado, Guilherme DP Soares, Carlos Ramires Neto, Kathleen Linn

**Affiliations:** 1 Univ Estadual Paulista (UNESP), Department of Veterinary Surgery and Anesthesiology, School of Veterinary Medicine and Animal Science, Botucatu, São Paulo, Brazil; 2 Univ Estadual Paulista (UNESP), Department of Surgery and Orthopedics, School of Medicine, Botucatu, São Paulo, Brazil; 3 University of Saskatchewan, Department of Small Animal Clinical Sciences, Western College of Veterinary Science, Saskatoon, Canada

## Abstract

**Background:**

The aim of this report was to describe the clinical signs, diagnostic approach, treatment and outcome in the case of a tiger with a deformity of the paw.

**Case presentation:**

A 1.5-year-old tiger (*Panthera tigris*) was presented with lameness of the left thoracic limb. A deformity involving the first and second metacarpal bones, and a soft tissue separation between the second and third metacarpal bones of the left front paw were observed. The second digit constantly struck the ground during locomotion. Based on the physical and radiographic evaluations, a diagnosis of ectrodactyly was made. A soft tissue reconstruction of the cleft with excision of both the second digit and distal portion of the second metacarpal bone was performed. Marked improvement of the locomotion was observed after surgical treatment, although the tiger showed a low degree of lameness probably associated with the discrepancy in length between the thoracic limbs.

**Conclusion:**

This report shows a rare deformity in an exotic feline that it is compatible to ectrodactyly. Reconstructive surgery of the cleft resulted in significant improvement of limb function.

## Background

There are several types of congenital hand anomalies in human patients and a variety of classification systems [1,2]. According to morphological classification polydactyly indicates the presence of extra digits, syndactyly is an abnormal linkage between adjacent digits, brachydactyly refers to short digits, macrodactyly denominates large digits, and ectrodactyly or oligodactyly denotes defective digits [1,3].

Ectrodactyly has been described in dogs and domestic cats but is considered rare [3-9]. In general, the anomaly in these species is unilateral and affects only the thoracic limb [4,6-13], but bilateral involvement also has been reported [10,14,15]. A radiographic study in dogs with ectrodactyly classified the defects based on the site of division of the longitudinal axis of the paw [10]. Axial separation between metacarpal bones; abnormal carpal, metacarpal and phalangeal bones; syndactyly; and separation between the radius and ulna are some of the radiological features reported [5,7,10,14]. In addition, elbow luxation has been associated with some ectrodactyly cases in dogs [5,7,10,12]. Clinical signs of deformity and lameness may progress with age, even if the malformation has been present since birth [3,7].

The purpose of the case reported here is to describe the clinical signs, diagnostic approach, treatment and outcome in the case of a tiger with a deformity of the paw.

## Case presentation

A 1.5-year-old, 150 kg, intact male tiger (*Panthera tigris*) was admitted to the Veterinary Hospital of the School of Veterinary Medicine and Animal Science (Unesp Botucatu) with a history of left thoracic limb lameness that had started to worsen approximately five months previous to admission. In addition, the tiger had avoided scratching with this paw. According to the owner, the tiger had a deformity in the left paw which had been noticed since birth, and this limb had always been lame. No treatment had been performed. He was the only cub born in a litter. Two other litters from the same parents showed no evidence of deformity. The tiger was kept in captivity in a large area with seven more tigers. The owner is authorized by federal agency to raise the tigers, and all animals are submitted to training program.

### A) Clinical examination and diagnosis

**Figure 1:**
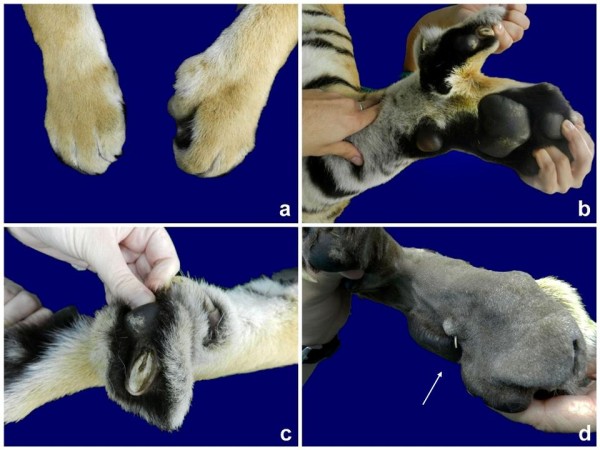
**Ectrodactyly deformity in the left front paw of a tiger.** Note the cleft between the second and third metacarpals on the dorsal (**a**) and palmar views (**b**), the claw worn down at the base on the second digit (**c**), and the extra claw (arrow) (**d**).

The body condition indicated an obese animal. A deformity involving the second and third digits of the left front paw was observed. During walking the tiger showed lameness of the left thoracic limb, but without reducing the weight bearing of the affected limb when standing or sitting (Additional file 1: Movie 1). However, the second digit constantly struck the ground during locomotion. No muscle atrophy was observed. For physical and radiographic examination, the tiger was premedicated with ketamine (3 mg/kg IM; Ketamin, Cristália, Brazil) and dexmedetomidine (2 μg/kg IM; Precedex, Abbott, Brazil) using a blow dart, and anesthesia was induced with propofol (1 mg/kg IV; Propovan, Cristália, Brazil) and maintained with isoflurane (Isoforine, Cristália, Brazil). No abnormality was detected during the flexion and extension of the thoracic limb joints. A soft tissue separation between the second and third metacarpal bones of the left front paw was observed (Figure 1a and 1b). The claw of the second digit was worn down at the base (Figure 1c).

**Figure 2:**
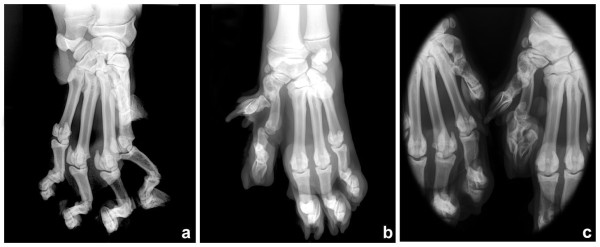
**Dorsopalmar radiographs of the right (a) and left (b) paws, and both paws (c) in a tiger with ectrodactyly.** Observe in the left paw (**b**) the separation of soft tissue between the second and third metacarpal bones, separation between second and third carpal bones, first metacarpal somewhat misshapen and fused to the proximal aspect of second metacarpal, and hypoplasia (shortened) of the second metacarpal bone with associated metacarpophalangeal luxation with the head of phalanx one malformed. Magnified image to compare the first and second digits from both the left and right paws (**c**).

Medio-lateral radiography of both shoulders and both radius and ulna were performed, and medio-lateral and dorsopalmar radiographic views of both paws (Figure 2) were obtained. Radiographic views of both shoulders showed a small bone fragment at the caudal border of the glenoid cavity on the left shoulder. Comparison of the lengths of both radii revealed the left to be approximately 2 cm shorter than the right. However, the radial and ulnar physes had a normal appearance. Radiographic findings of the left paw included separation of soft tissue between the second and third metacarpal bones, separation between second and third carpal bones, the first metacarpal somewhat misshapen and fused to the proximal aspect of second metacarpal, and hypoplasia (shortening) of the second metacarpal bone with associated metacarpophalangeal luxation with the head of phalanx one malformed (Figure 2b). The first digit of the left paw had 2 phalanges as well as normal paw (Figure 2c).

**Figure 3:**
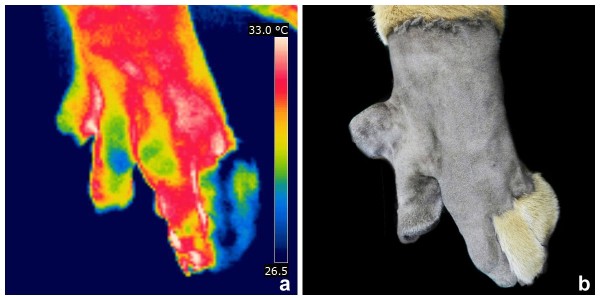
**Thermal image (a) and macroscopic aspect (b) of the left front paw before surgery in a tiger with ectrodactyly.** Observe the difference of thermal pattern between the second and third digits.

Infrared thermography **(**Infra CamTM; FLIR Systems Inc.), accomplished by the software ThermaCAM Quick Report, was used to evaluate both paws and shoulders. Mean temperatures of the second and third digits of the left paw were 29°C and 31°C, respectively (Figure 3). No temperature differences were observed between right and left shoulder.

Based on the findings from the physical and radiographic examinations, a diagnosis of ectrodactyly was made.

### B) Treatment and outcome

**Figure 4:**
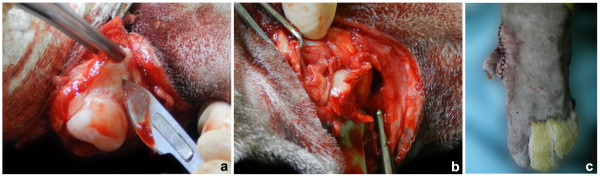
**Observe the left second metacarpal bone after disarticulation with the proximal phalanx (a), and after removing its distal portion (b) during the surgical reconstruction of ectrodactyly in a tiger.** Aspect of the left front paw immediately after surgery (**c**).

**Figure 5:**
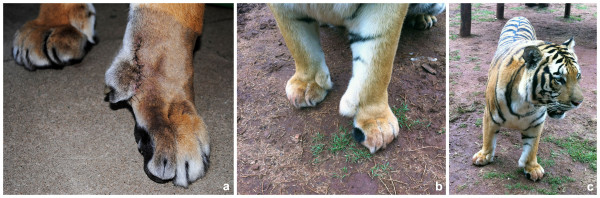
**Observe the left front paws at 10 days (a) and 9 months (b) after surgery.** Notice the tiger in a standing position 9 months postoperatively (**c**).

After receiving the owner’s consent, the surgical procedure was performed. Under general isoflurane (Isoforine, Cristália, Brazil) anesthesia, the tiger was positioned in right lateral recumbency and the left paw area was clipped and aseptically prepared for surgery. After clipping it was possible to identify one extra claw, which was located on the mediolateral region of the third metacarpal bone (Figure 1d). Two semicircular incisions were made dorsally and palmarly around the second digit metacarpophalangeal joint. The subcutaneous tissue was dissected, the arterial supply was ligated, the flexor and extensor tendons were transected, and the phalanges were removed by disarticulation between the metacarpal bone and proximal phalanx (Figure 4a). The distal portion of the metacarpal bone was removed using a roungeur (Figure 4b). A triangle of excess skin was removed dorsally. The retracted soft tissue was apposed with simple interrupted sutures and the subcutaneous tissue with a simple continuous pattern using sutures of 2-0 and 3-0 polyglactin, respectively. The skin incision was closed using simple interrupted sutures of monofilament 2-0 nylon (Figure 4c). Ceftriaxone (30 mg/kg IV; Ceftriona, Novafarma, Brazil) was administered during the surgery. Meloxicam (0.1 mg/kg orally q24h; Maxicam, Ouro Fino, Brazil) was administered immediately postoperatively and for two days after surgery. No complications were observed after surgery. Ten days after surgery the tiger was discharged (Figure 5a) (Additional file 2: Movie 2). Marked improvement of the locomotion was observed. An additional follow-up at 9 months after surgery (Figure 5b and 5c) found a good functional outcome for the tiger despite a low degree of lameness. According to the owner, the tiger returned to scratching the trees with the operated paw.

## Discussion

In dogs and domestic cats, ectrodactyly has been considered a highly heterogeneous disorder with different sites of soft tissue separation and a variety of abnormalities [7,8,10]. The radiographic and macroscopic features noted in this tiger were compatible with some reported cases of ectrodactyly [7,10]. A study of 14 dogs with ectrodactyly found that the soft tissue separation extended up to the proximal metacarpal level in 85.7% of the cases and mainly between metacarpal bones one and two [10]. The same was noted in the present case, but the separation occurred between metacarpal bones two and three. Absence or hypoplasia of metacarpal and carpal bones may be present in cases of ectrodactyly [10]. In the present case, the second metacarpal bone was also hypoplastic. The luxation between this bone and the proximal phalanx could be congenital since the phalanx was malformed. There was also a traumatic component since the tiger was constantly striking his second digit on the ground. Differences in bone length such as decreased or increased length of both radius and ulna, or decreased ulna or increased radius have also been described [10]. In the present case both the radius and ulna were shorter in the affected limb. The radius and ulna physes appeared normal, implying that premature closure was not the cause of the shortened bones.

A supernumerary digit constituted only by the distal phalanx and connected to the fourth digit by cutaneous tissue was observed in a dog with ectrodactyly [11]. In the present case an extra claw was observed and presented no osseous connection to the third metacarpal bone.

In the majority of the reported canine ectrodactyly cases, the cause was unknown [5,7,8,10,15]. However, in domestic cats a mode of inheritance due to a heterozygous gene with variable expression has been demonstrated [14]. Except for hemivertebra [15], other detectable congenital abnormalities have not been found in most of the reported cases of ectrodactyly in dogs and cats [4-10,12,13]. In the present case, the tiger was the first of his species to show such a deformity, and no other abnormality was observed.

There is no specific management or treatment strategy for congenital anomalies, but the primary goals are to prevent progression of the condition and to improve quality of life. In case of minor lesions of ectrodactyly that do not affect limb function no treatment is required; however, reconstruction is necessary in severe cases [5,7-9,12]. In the present case the malposition of the digit was inducing lameness, because of the constant trauma of the digit on the ground as confirmed by the worn nail and luxation between the metacarpal bone and proximal phalanx. In addition, infrared thermography showed difference in heat radiation between the second and third digits. Since the second digit had decreased heat radiation, the lesion may be characterized as not inflammatory. Infrared thermography can be helpful to detect the source of lameness in wild animals, as observed in the present case [16]. Furthermore, no temperature differences were observed between right and left shoulder. Probably the small bone fragment at the caudal border of the glenoid cavity on the left shoulder was an incidental incomplete ossification of the glenoid rim.

Ulnar osteotomy or ostectomy, partial or pancarpal arthrodesis, use of orthopedic wire around adjacent bones, bone lengthening and soft tissue reconstruction are some procedures reported in dogs for treatment of ectrodactyly [5,7-9,12]. Surgical decision making and selection of the appropriate procedure depends on the species being treated, the type of deformity, the timing of surgery and the possible risk of physeal arrest [7,9]. A few cases have been managed by amputation [6,13]. In the present study, a soft tissue reconstruction of the cleft with excision of both the second digit and a portion of the second metacarpal bone was considered the best option. The third and fourth digits are considered the most important in dogs and cats because they are the primary weight-bearing digits, and no lameness is expected as a result of second digit removal [17]. Therefore, the low degree of lameness still present in the tiger is probably associated with the discrepancy in length between the thoracic limbs.

## Conclusions

This report shows a rare deformity in an exotic feline that it is compatible to ectrodactyly. Reconstructive surgery of the cleft resulted in significant improvement of limb function.

## Consent

Consent was obtained from the owner of the tiger for publication of this case report and any accompanying images.

## Authors' contributions

SCR and RSV performed the surgical procedure and prepared the manuscript, VMVM performed the radiographic examination, CRRN performed the thermographic examination, CRT and GDPS carried out the clinical examination and prepared the tiger to surgery, KL reviewed the literature. All authors have read and approved the manuscript.

## Supplementary Material

Additional file 1 **Movie 1.** Observe the lameness of the left thoracic limb in a tiger with ectrodactyly in the left front paw before the surgery. Click here for file

Additional file 2 **Movie 2.** Observe the marked improvement of the locomotion after 10 days after surgery. Click here for file

## References

[B1] Ogino T Clinical features and teratogenic mechanisms of congenital absence of digits Develop Growth Differ 2007 49 523 531 10.1111/j.1440-169X.2007.00939.x 17555519

[B2] Chung MS Congenital differences of the upper extremity: classification and treatment principles Clin Orthop Surg 2011 3 172 177 10.4055/cios.2011.3.3.172 21909463 PMC3162196

[B3] Towle HAM Breur GJ Dystoses of the canine and feline appendicular skeleton J Am Vet Med Assoc 2004 225 1685 1692 10.2460/javma.2004.225.1685 15626218

[B4] Schneck GW Two cases of congenital malformation (peromelus ascelus and ectrodactyly) in cats Vet Med Small Anim Clin 1974 69 1025 1026 4495778

[B5] Montgomery M Tomlinson J Two cases of ectrodactyly and congenital elbow luxations in the dog J Am Anim Hosp Assoc 1985 21 781 785

[B6] Frey M Williams J What is your diagnosis? (Ectrodactyly in a Chow Chow dog) J Am Vet Med Assoc 1995 206 619 620 7744678

[B7] Innes JF McKee WM Mitchell RAS Lascelles BDX Johnson KA Surgical reconstruction of ectrodactyly deformity in four dogs Vet Comp Orthop Traumatol 2001 14 201 209

[B8] Barrand KR Ectrodactyly in a West Highland white terrier J Small Anim Pract 2004 45 315 318 10.1111/j.1748-5827.2004.tb00243.x 15206479

[B9] Harasen G Surgical management of ectrodactyly in a Siberian husky Can Vet J 2010 51 421 424 20592835 PMC2839836

[B10] Carrig CB Wortman JA Morris EL Blevins WE Root CR Hanlon GF Suter PF Ectrodactyly (split-hand deformity) in the dog Vet Radiol Ultrasound 1981 22 123 144 10.1111/j.1740-8261.1981.tb01363.x

[B11] Oliveira D Artoni SMB Ectrodactilia em cão (Canis domestica) Cienc Rural 2002 32 1063 1065 10.1590/S0103-84782002000600023

[B12] Ferreira MP Alievi MM Beck CAC Voll J Muccillo MS Gomes C Ectrodactyly in dog: case report Arq Bras Med Vet Zootec 2007 59 910 913 10.1590/S0102-09352007000400015

[B13] Mehrjerdi HK Hayati F Sardari K Mirshahi A Gachpaz S Ectrodactyly in a mix breed dog Iranian J Vet Surg 2008 3 87 91

[B14] Searle A Hereditary split-hand in the domestic cat Ann Eugen 1953 17 279 282 13041028

[B15] Carvallo FR Domínguez AS Morales PC Bilateral ectrodactyly and spinal deformation in a mixed-breed dog Can Vet J 2010 51 47 49 10.4141/cjas72-005 PMC3003574 21461206

[B16] Hilsberg-Merz S Fowler ME, Miller RE Infrared thermography in zoo and wild animals Zoo and Wild Animal Medicine Current Therapy 2008 Elsevier, St. Louis, Saunders 20 32

[B17] Hedlund C Fossum TW Surgery of the digits and footpads Small Animal Surgery 2007 Elsevier, St. Louis, Mosby 250 259

